# Effect of Cellulose Nanocrystals from Different Lignocellulosic Residues to Chitosan/Glycerol Films

**DOI:** 10.3390/polym11040658

**Published:** 2019-04-10

**Authors:** Marina Reis de Andrade, Tatiana Barreto Rocha Nery, Taynã Isis de Santana e Santana, Ingrid Lessa Leal, Letícia Alencar Pereira Rodrigues, João Henrique de Oliveira Reis, Janice Izabel Druzian, Bruna Aparecida Souza Machado

**Affiliations:** 1 Department of Materials, University Center SENAI CIMATEC, National Service of Industrial Learning, Salvador 41650-010, Brazil; marina.andrade@fbter.org.br (M.R.d.A.); tayisis.santana@gmail.com (T.I.d.S.e.S.); 2 Department of Food and Beverages, National Service of Industrial Learning, Applied Research Laboratory of Biotechnology and Food, University Center SENAI CIMATEC; Salvador 41650-010, Brazil; tatianabr@fieb.org.br (T.B.R.N.); ingrid.leal@fieb.org.br (I.L.L.); leticiap@fieb.org.br (L.A.P.R.); 3 Biotechnology, Pharmacy Faculty, Federal University of Bahia; Salvador 40170-115, Brazil; jhonhyba47@hotmail.com (J.H.d.O.R.); druzian@ufba.br (J.I.D.); 4 University Center SENAI CIMATEC, National Service of Industrial Learning, Laboratory of Pharmaceutical’s Formulations, Health Institute of Technologies (ITS CIMATEC), Salvador 41650-010, Brazil

**Keywords:** films, nanocellulose, nanocrystals, biodegradable packaging

## Abstract

Interest in nanocellulose obtained from natural resources has grown, mainly due to the characteristics that these materials provide when incorporated in biodegradable films as an alternative for the improvement of the properties of nanocomposites. The main purpose of this work was to investigate the effect of the incorporation of nanocellulose obtained from different fibers (corncob, corn husk, coconut shell, and wheat bran) into the chitosan/glycerol films. The nanocellulose were obtained through acid hydrolysis. The properties of the different nanobiocomposites were comparatively evaluated, including their barrier and mechanical properties. The nanocrystals obtained for coconut shell (CS), corn husk (CH), and corncob (CC) presented a length/diameter ratio of 40.18, 40.86, and 32.19, respectively. Wheat bran (WB) was not considered an interesting source of nanocrystals, which may be justified due to the low percentage of cellulose. Significant differences were observed in the properties of the films studied. The water activity varied from 0.601 (WB Film) to 0.658 (CH Film) and the moisture content from 15.13 (CS Film) to 20.86 (WB Film). The highest values for tensile strength were presented for CC (11.43 MPa) and CS (11.38 MPa) films, and this propriety was significantly increased by nanocellulose addition. The results showed that the source of the nanocrystal determined the properties of the chitosan/glycerol films.

## 1. Introduction

With the advancement of nanotechnology and nanoscience, materials are modulated in their technologies, generating new technologies to incorporate to the needs of the current society [1,2,3,4,5]. In this context, cellulose nanocrystals are being used to improve the mechanical and barrier properties of chitosan films and make their commercialization viable [6,7,8,9,10,11]. Nanocellulose or cellulose nanocrystals are the crystalline domains of cellulosic sources, obtained through acid hydrolysis, having characteristics of high rigidity, high crystallinity, and nanometric size [6,7,8,9,10]. The cellulose nanocrystals have been the object of several studies, since they present great potential of application as reinforcement in polymer matrices [12,13]. Cellulose nanocrystal is known as the most appropriate and efficient reinforcement additive due to its renewability, excellent mechanical properties, and economic cost [14], and can be obtained from waste, further improving the cost benefit.

Among the nanoreinforced materials, the application of the vegetal fibers stands out, since these have ample availability in almost all the countries, being usually designated as lignocellulosic materials [15,16,17]. Some fibers are found spontaneously in nature, while others are derived from agricultural activity and from waste generated mainly by agroindustry. Studies evaluate the application of nanocrystals obtained by several fibers in polymeric systems, for example, barley straw and husk in poly(vinyl alcohol) (PVA) blended with natural chitosan (CH) nanocomposites [18], pine cones in a biodegradable poly(3-hydroxybutyrate)/poly(ε-caprolactone) (PHB/PCL) [19], and sunflower stalks on wheat gluten bionanocomposites [20]. The high availability of lignocellulosic fibers, coupled with the need for a renewable source for the production of polymers, represents a great opportunity for technological advances that add value to the products or residues of the agroindustry and, at the same time, act in the fixation of carbon in nature [21,22,23,24]. This implies helping to reduce the emission of CO_2_ into the atmosphere during the production cycle, increasing the economic potential of agribusiness due to the possibility of trading carbon credits in the production chain [25,26,27,28,29].

In relation to the development of new materials, there is also the growth of technologies using polymers from renewable sources for diverse applications. These materials have been important for the advancement of the sciences, and have several advantages such as being easily obtainable, biocompatible, and biodegradable [30,31]. The choice of material to be used in the formulation of the films is very important, as they will depend on the interactions between the components of the material, which may interfere with the barrier properties, mechanical properties, and the physical aspects of the films [32]. 

Several biopolymers such as polysaccharides, proteins, and lipids have been used as polymer matrices for the development of biodegradable packaging due to their availability, renewability, low cost, respect for the environment, and biodegradability. Among these, chitosan is considered favorable for the development of biocomposites [33,34], and based on production volumes, is the second most abundant polymer after cellulose [35,36]. Chitosan is a natural polysaccharide derived from chitin, and although the most important sources are commercial shellfish carapaces, studies indicate that this element can be found in insects, mollusks, and fungal cell walls [37]. The bioactivity of this material has aroused interest in the application as a packaging film due to its ability to form flexible and resistant films with efficient oxygen barrier and antimicrobial activity [6,7,9,38,39]. In addition, the use of chitosan in the field of biomedicine has been reported for its various important pharmacological properties and its role in tissue engineering, regenerative medicine, scaffold, and drug delivery systems is also well documented [40]. 

Chitosan based films are biocompatible and biodegradable, with excellent mechanical strength and cost effectiveness [41]. Previous studies have shown the efficiency of the incorporation of nanocrystals obtained from different sources into chitosan films, contributing to increase the mechanical and barrier properties of these materials [42,43,44]. Azeredo et al. [45] evaluated the effect of different concentrations of nanocellulose on tensile properties and water vapor permeability of chitosan films. Pereda et al. [46] demonstrated that the combined use of cellulose nanoparticles and olive oil proved to be an efficient method to reduce the inherently high water vapor permeability of plasticized chitosan films, improving their tensile behavior at the same time.

The films obtained by natural polymers are poorly flexible and brittle, thus, it becomes necessary to add plasticizer to the polymer matrix to improve its flexibility characteristics. Plasticizers reduce the interactions between adjacent molecules, increasing film flexibility [47]. For application of a plasticizer, it is extremely important that it is suitably compatible with the polymer used and the definition of proportionality between the components in order to tailor the final composition to a given application [48]. Several plasticizers are used in the preparation of biodegradable films and coatings, including mono-, di- and oligosaccharides (glucose, sucrose); polyols (glycerol, sorbitol, derivatives of glycerol); and lipids (saturated fatty acids, monoglycerides and ester derivatives, phospholipids and surfactants) [49]. The use of plasticizers in these films allows a greater percentage of elongation and adaptation of the matrix in the structure [50]. Glycerol is currently one of the plasticizers most used in the development of biodegradable films, which has caused scientific impact since this polyalcohol is a byproduct generated from the biodiesel chain, which is expanding worldwide [51], and is thus a low-cost and high-availability material.

With this background, the objective of this work was the extraction of cellulose nanocrystals from four different lignocellulosic fibers, considered as byproducts of the agro-food industries (Corncob (CC), corn husk (CH), wheat bran (WB) and coconut shell (CS)) and the investigation of the influence of the incorporation of these nanoparticles on the physical, barrier, and mechanical properties in chitosan biofilms. The main focus of the study is to identify sources of residues for the production of cellulose nanocrystals and, consequently, to evaluate the behavior of the inclusion of these nanoparticles in films using chitosan as a polymer matrix. Chitosan is identified as a highly attractive biomaterial for film owing to its properties seen in previous reports. Chitosan can be easily incorporated into gels, membranes, beads, and scaffolds, and these forms provide a wide variety of biomedical applications and food packaging.

## 2. Materials and Methods 

For extraction of the cellulose nanocrystals were used corncob (CC) and corn husk (CH) bought in local commerce in Salvador, Bahia, Brazil, wheat bran (WB) provided by a local wheat mill, and coconut shell (CS) donated by Frisbraztech (Conde, Bahia, Brazil). The films were produced with chitosan (Sigma-Aldrich, Saint Louis, MO, USA, Cas Number: 9012-76-4, with a degree of deacetylation ≥ 75%), and the glycerol purchased from Synth (São Paulo, Brazil).

### 2.1. Characterization of the Fibers

The natural fibers were characterized as moisture, water activity, ash content, and crude fiber content.

The moisture content was determined using an infrared-heated scale (Shimadzu, MOC-120H, Kyoto, Japan) with the intensity of the emitted radiation set so that the sample would reach 105 °C. Measurements of water activity were performed using a Decagon (Novasina^®^, Lab Master aw, Neuheimstrasse, Switzerland) at temperature of 25 °C. The ash content was determined using an muffle Fornitec, and crude fiber content (lignin, hemicellulose and cellulose) in Ankom A200 Fiber Analyzer (New York, NY, USA), by the FDA (Acid Detergent Fiber) and NDF (Neutral Detergent Fiber), according to the methodology proposed by Van-Soest, Robertson and Lewis [52].

### 2.2. Extraction of Cellulose from Fibers 

The extraction of the cellulose pulp was performed based on the works of Samir et al. [53] and Machado et al. [12]. The selected materials (CC, CH, WB and CS) were previously dried at 60 °C for 3 h to remove excess moisture and ground in a blender to obtain a fine powder. The crushed fibers (30 g) were washed in 1200 mL of 2% NaOH solution and at 80 °C for a period of 4 h under constant stirring. The resulting solutions were filtered and washed with water to obtain a pulp. The washing process was repeated three times for complete removal of the water soluble agents and obtaining the cellulose pulp. After washing, the process of delignification and bleaching of pulps was carried out, using 300 mL of sodium hypochlorite (1.7%) and 300 mL of a buffer solution. The resulting solution was placed under constant stirring at a temperature of 80 °C for 6 h (TE-394/2, Tecnal, São Paulo, Brazil), filtered and oven dried (40 °C) to obtain the cellulose pulp of each fiber.

### 2.3. Preparation of Cellulose Nanocrystals

The cellulose nanocrystals were prepared by acid hydrolysis using 45% H_2_SO_4_ [12,38]. Briefly, 12 mL/g of cellulose was subjected to constant stirring for period of 1 h, and temperature between 50 and 55 °C. After acid hydrolysis, the dispersions were cooled to 30 °C and the volume was completed to 40 mL in Falcon tubes. The tubes were centrifuged for 10 minutes at 4400 rpm and a temperature of 10 °C (Sigma 2-16KHL, Osterode am Harz, Germany) in order to separate the crystals in suspension. Centrifugation was repeated 4 to 6 times for better separation results.

Then the suspensions were subjected to dialysis using cellulose membranes (D9777-100 FTO, 12.000 Da cut off, Sigma-Aldrich, Saint Louis, MO, USA), and after reaching the pH between 6 to 7, the samples were placed in an ultrasonic bath (RMS, Quimis, São Paulo, Brazil) with a power of 200 W, frequency of 60 kHz, and a temperature of 25°C for 5 min for the dispersion of the nanocrystals.

### 2.4. Production of Films (Nanobiocomposites)

The films with nanoparticles extracted from different fibers were produced by the casting method, according to the methodology proposed by Yassue–Cordeiro et al. [31] and de Souza [54]. Chitosan (1.5%, g/100g) dissolved in glacial acetic acid (1.0%, g/100g) and glycerol (0.15%, g/100g) was used as the plasticizing agent. To this mixture was added 5% (w/v) cellulose nanocrystals (a filmogenic solution for each fiber) under constant shaking in Shaker Incubator (MA420, Marconi, São Paulo, Brazil) at 40 °C for 24 h (Table 1). After homogenization, 40 g of the solution was transferred to Petri dishes and subjected to dehydration in an air circulation oven (35 ± 2 °C) (Q314M, Quimis, São Paulo, Brazil) for 20 h. Other film was produced without the addition of cellulose nanocrystals for use as control. The films obtained were packed in a vacuum desiccator containing saturated sodium chloride solution (TE-3950, Tecnal, São Paulo, Brazil).

### 2.5. Characterization of Cellulose Nanocrystals

The concentration of nanocrystalline cellulose in the suspensions was determined by gravimetric analysis. An aliquot with a known volume was dried at 40 °C for 24 h in an air circulation oven (TE-394/2, Tecnal, São Paulo, Brazil). The birefringence of the nanocellulose suspensions was determined by the methodology proposed by Flauzino et al. [55], an aliquot of aqueous suspension of the nanocrystals (5 x 10^−3^ g.mL^−1^) was placed in a glass test tube, this tube was placed in front of a polarized light source, and it was then photographed with a camera equipped with a filter of polarized light. Cellulose nanocrystalline dispersions for all fibers were analyzed by transmission electron microscopy (TEM), in order to determine the length of the fibers (L), width (D), and aspect ratio (L/D) and indicate the state of aggregation of the crystals. Measurements were made directly from the micrographs using Image Tool 6.3 (Media Cybernetics, Rockville, USA) with 30 measurements to determine mean and standard deviation values according to the methodology proposed by Machado et al. [12].

### 2.6. Characterization of Films—Determination of Thickness and Mechanical Properties

The thickness of the films and control were evaluated in random positions using digital micrometer (Digimess, São Paulo, Brazil) with resolution of 0.001 mm. The tensile tests were performed using a Brookfield (Braseq CT310K, Middleboro, MA, USA), with a maximum load of 10 KN, with a speed of 0.5 mm s^−1^, a temperature of 25 °C, trigger load of 7 g, test probe tip of TA3/100 and TA / TPB device [13,56]. Tensile tests were performed on six specimens with dimensions of 80 mm in length and 25 mm wide for each sample.

### 2.7. Statistical Analysis 

The results of this study were expressed as the mean ± standard deviation (sd) (n = 3). The statistical analysis of the results was performed using the Statistica^®^ 6.0 software from StatSoft (Tulsa, Hamburg, Germany). The results were treated by the Tukey test to identify if the changes in the parameters evaluated were significant at the 95% level of significance.

## 3. Results

### 3.1. Characterization of Fibers 

The composition and structure of lignocellulosic biomass have great influence on the nature and yields of the hydrolysis processes. The moisture content (%), the water activity (a_w_), and the ash content (%) of the fibers obtained from corn cob (CC), corn husk (CH), wheat bran (WB), and coconut shell (CS) are shown in Table 2.

Table 3 shows the cellulose, hemicellulose and lignin contents of the fibers studied. Each component of the lignocellulosic fibers is responsible for different functions, so different levels of these components influence the properties of the nanocellulose fibers obtained [38,57,58].

### 3.2. Characterization of Cellulose Nanocrystals

**Figure 1:**
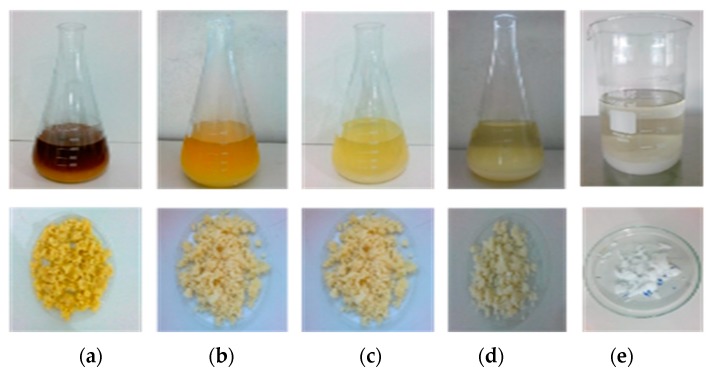
Cellulose pulp obtained by corncob (CC): (**a**) first wash with NaOH; (**b**) second washing; (**c**) third washing; (**d**) fourth washing; (**e**) bleaching step.

Figure 1 shows the four steps of washing the fiber with NaOH for the process of extracting cellulose from the corncob, as an example, until the bleaching stage.

For each 30 g of fiber submitted to the washing and bleaching process (Figure 1), different yields of cellulose pulp were obtained, and these results are presented in Table 4. The concentration of nanocrystals (g.10mL^−1^) is a parameter for the evaluation of the nanocellulose dispersion, indicating if it needs a higher concentration for use in the production of the films.

**Figure 2:**
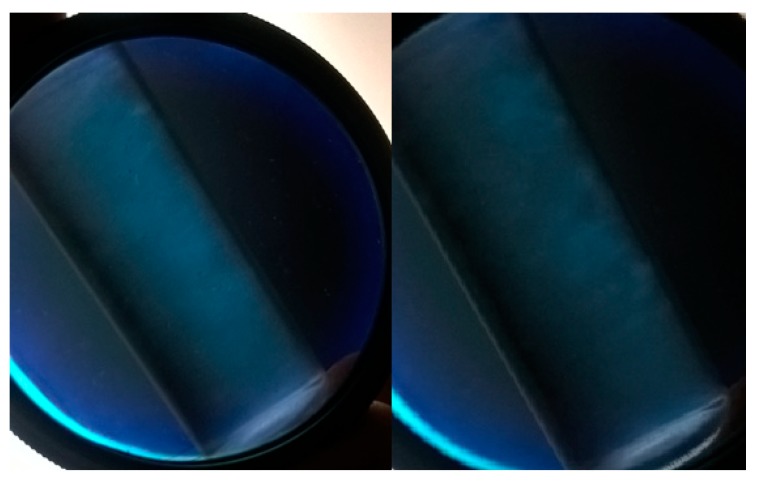
Phenomenon of birefringence observed through a polarized lens after dispersion of cellulose nanocrystals extracted from corncob (CC).

The existence of nanocrystals can be proven by birefringence analysis and microscopy. According to Pereira et al. [59], flow birefringence results from the alignment of nanoparticles and indicates the existence of isolated nanocrystals in the dispersion. As the cellulose nanocrystals are rigid rod-like particles, they have a strong tendency to align a vector director and increase in relation to particle size / diameter. As a result of the strong birefringence of the native cellulose, this rod alignment creates a macroscopic birefringence that can be directly observed though cross polarizers [60]. The suspensions (CH, CC, CS and WB) were analyzed from a polarizing lens. In the birefringence analysis, the CC nanocrystals dispersion was the one that presented the nematic phase liquid crystals (N) clearly to prove the existence of the crystals (Figure 2), CH and CS presented nematic phase with less clarity, and the WB did not present nematic phase. This information is in agreement with the results presented in Table 5.

Table 5 presents the mean values of the width (D), length (L), and the L/D ratio of the crystals.

**Figure 3:**
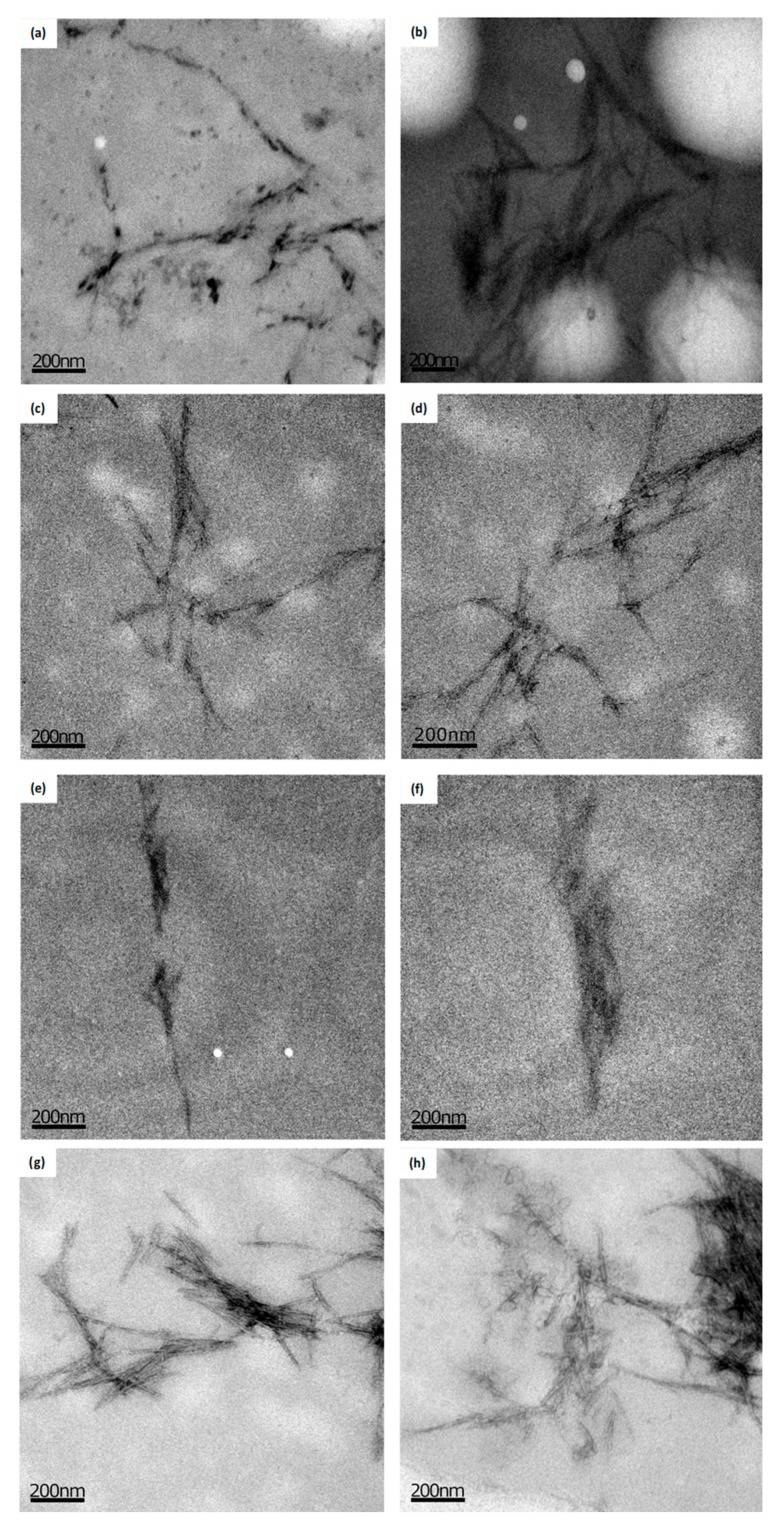
Cellulose nanocrystals obtained by Transmission Electron Microscopy (TEM) (PTA contrast and uranyl). (**a**) and (**b**) Corn husks; (**c**) and (**d**) Corncob; (**e**) and (**f**) Wheat bran; (**g**) and (**h**) Coconut shell (Scale: 200 nm).

CC, CH, and CS presented positive results for TEM analysis, where it was possible to visualize the aggregate crystals in a needle format (Figure 3). It was not possible to visualize crystal formation for wheat fiber, indicating that they are not present in a considerable amount. 

The process conditions, whether concerning fiber preparation or hydrolysis for whisker isolation, affect the morphological characteristics of these nanomaterials. The acid used for hydrolysis may affect the characteristics of whisker dispersion in an aqueous system. The effect of reaction time and acid-wood pulp ratio on the properties and behavior of the whisker suspension, obtained by sulfuric acid hydrolysis, was observed that shorter whiskers, less variable in length, were obtained in longer reaction times [61]. Beck-Candanedo et al. [61] also found out that an increase in the acid-pulp ratio also leads to whiskers with reduced dimensions. 

### 3.3. Production and Characterization of Films

**Figure 4:**
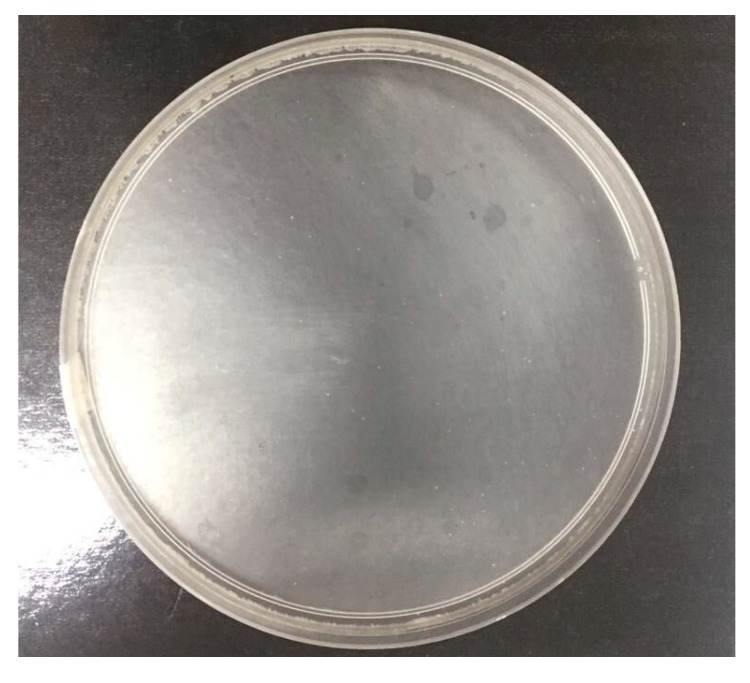
Physical appearance of CS film.

The films produced from the formulations described in Table 1, using different types of nanocrystals, as well as the control film, were analyzed to determine their physical and barrier properties (water activity (a_w_), moisture content (M), total solids (TS), and thickness (*t*)) and their mechanical properties (tensile strength (σ) and strain (ε)), described in Table 6. Figure 4 shows the physical appearance of CS film. 

## 4. Discussion

Among the fibers, CS had the highest results to moisture (88.7% ± 0.07), water activity (0.97 ± 0.05), and ash (5.37% ± 0.10), the WB presented the smaller moisture content (12.4% ± 0.60), and water activity (0.640 ± 0.03) (Table 2). The moisture content of the fibers, as well as the storage conditions and the time, can interfere in the degree of crystallinity of the cellulose, and consequently, in obtaining the nanocrystals [15,29,54]. The CH and CC fibers analyzed in this study were collected from ears of green and not dry corn. For this reason, the values found for moisture and water activity were higher than those found in the literature. Corn husk presented higher moisture than that presented by Reference [62] (12.96%) and near ash content (1.52%), which can be explained mainly by the origin of the fiber and its level of maturation. Ziglio et al. [63] found moisture values of 8.9% for cob corn [63]. The results of moisture found for wheat bran are similar to those presented by De Lima Dantas et al. [64], 11.59%, however, the ash value was lower (0.62%). Differences can be justified by the wheat variety, the maturation stage of the samples, and the producer region.

The results in Table 3 show the differences in fiber composition of the different residues studied. The CC fiber showed the highest values of cellulose (52.99% ± 1.79) followed by the CS fiber (47.16% ± 1.24). The CC fiber presented the highest hemicellulose content while the CS fiber had the highest lignin content (30.71 ± 0.21). Souza et al. [65] found values of 37.6% cellulose, 34.5% hemicellulose, and 12.6% lignin for corn husk, and 31.7% cellulose, 34.7% hemicellulose, and 20.3% lignin for corn cob. In the literature, different values of cellulose (56.8%) and lignin (29.8%) were presented for coconut shell [66]. 

In their study, Merali et al. [67] found values of 18.5% cellulose, 54.8% hemicellulose, and 10.8 lignin in pretreated WB hydrothermally. These values may be associated to the exchange rate in the fiber, in the region where it was extracted and its botanical varieties. Mendes et al. [68] found values of 33–40% cellulose, 33–40% hemicellulose, and 2–16% lignin in CH residue samples. The values found by the authors show the large range of these constituents in the samples, which can be attributed to maize variety and harvest period.

The importance of determining cellulose at the fibers is that being a polysaccharide made of repeating beta 1,4-glycosidic bonds, it is characterized by having intercalated arrangements of highly ordered (crystalline) and amorphous (disordered) [69]. Thus, the isolation and obtaining of nanocrystals from lignocellulosic fibers depend directly on the proportion of these crystalline regions, as well as the lignin and hemicellulose content, since they interfere in the extraction process, the realization of pretreatment for removal of these components being necessary in many cases [28,31]. 

The visualization of the suspension of the cellulose nanocrystals obtained from the fibers studied using polarizers revealed a nematic phase, which was directly produced by light birefringence. This result was also important to confirm the presence of nanocrystals (except for WB) and is considered an important analysis to evaluate nanocrystals dispersion (Figure 2). Cerqueira et al. [13] and Alves et al. [17] similarly used crossed polarizers to visualize the birefringence phenomenon in suspension of cellulose nanocrystals obtained from coconut and eucalyptus, respectively. The nematic liquid crystal phase combines long-range orientational order with regular liquid-like short-range positional order. In a nematic CNC suspension, the nanocrystals align preferentially with their long axes along a common direction [70]. Perhaps there is a correlation among nematic phase (preferential orientation of the nanocrystals), refractive effects, and size of the CNC. In this study, a positive correlation was observed regarding the size of the nanocrystals obtained and the presence of birefringence. For example, the best nematic phase was presented by CC, which also has the highest L and D, and consequently, lower L/D ratio.

The morphological analysis is of great importance in determining the size and the state of agglomeration, considering that the source of cellulose as well as the technique used for the hydrolysis of the amorphous structure influence the size and the final properties of the nanoparticles [71,72]. Table 5 presents the mean values of the width (D), length (L), and the L/D ratio of the crystals. It is observed that the CC presented higher values of L and D and a lower L/D ratio. The values of (L) are in agreement with the literature [57,73] indicating a great potential of use of this fiber as reinforcement for bionanocomposites, as demonstrated in other studies. Similar results were reported by Machado et al. [74] (L = 98–430 nm, D = 6 nm e L/D = 38.9 ± 4.7), Rosa et al. [75] (L = 197 nm, D = 5.8 nm e L/D = 39), and Sarwar et al. [76] (240–280 nm).

Oliveira [77] notes that the resulting nanoparticle dimensions depend on the cellulose source and the hydrolysis process. Smaller nanoparticle diameters may be associated with higher amounts of hemicellulose present in the fiber structure, which would limit the organization of cellulose chains [78,79]. In this study, the CC fiber presented the highest concentration of cellulose and, consequently, presented the lowest L/D ratio for the nanocrystals obtained. The L/D ratio ranged from 32.19 to 40.86, being in agreement with other studies that presented values between 18.2 and 75.4 nm [13,79]. Some authors [11,80,81] found similar dimensions for the coconut shell. In this range, the crystals have great potential to be used as reinforcement in biodegradable films.

The film made with CH cellulose nanocrystals had a higher value of water activity (0.658), while the others presented approximate values of 0.600. The reduction of free water in packages for food products has the consequence of reducing the growth of microorganisms, avoiding undesirable chemical changes in the storage of the products [82,83,84,85]. Products that have water activity values lower than 0.600 are relatively protected against microbial contamination, whereas the proliferation of specific microorganisms can occur with water activity values above 0.600 [86,87,88]. Associated with the amount of free water of the product, the moisture of the film, which favors or inhibits proportionally the proliferation of microorganisms, is also evaluated [7,89].

The films produced with cellulose nanocrystals of CC and CS presented lower moisture content (18.32 ± 0.90 and 15.13 ± 0.01) in relation to films produced with WB and CH nanocrystals (20.86 and 20.24). In relation to the total solids, an increase is observed for the CC and CS, indicating that the amount of nonvolatile or water insoluble particles is slightly higher.

The thickness of the films (Table 6) was lower than the control film, maintaining unchanged barrier properties. Because it is a manual process, the standardization of the fluid distribution and the drying process are difficult. In a study, Hänninen et al. [90], observed that by adding a birch cellulose nanofiber, the thickness doubled compared to the control film containing only chitosan.

For this work, it was also observed that the incorporation of the nanocellulose dispersion, from the various fibers, to the film plasticized with glycerol, resulted in the improvement of the mechanical properties of the formulations studied. The tensile strength values (σ) varied between 4.08 MPa (control) to 11.38 MPa (CS Film) and 11.43 MPa (CC Film), presenting significant differences among the samples evaluated (except to CS and CC). The biofilms with the CS and CC nanocrystals dispersion presented a higher tensile strength at the rupture, indicating that a greater force was required for the rupture of the films, suggesting that there was an increase of the resistance. Regarding the deformation, the values found ranged from 115.9% (control) to 274.2% (CS film), and the WB film (110.0% ± 90.9) had a lower percentage of the evaluated fibers, with values close to the control. The lowest results in relation to the mechanical properties for the WB film may be justified due to the lowest concentration of cellulose in the fiber, and consequently the lowest efficiency to obtain the nanocrystals. This was also confirmed because of the impossibility of determining the size of the nanocrystals obtained by TEM (due to low concentration and formation of few clusters) as well as, no nematic phase formation. Benini (2011) [91] incorporated high impact polystyrene (HIPS) coconut fibers as a thermoplastic matrix, and the maximum stress and Young modulus were 23.7 MPa and 3.0 MPa for composites containing 30% of fibers.

The effect of surface and dispersion characteristics of the whiskers used as reinforcement material in a matrix with polypropylene was investigated by Ljungberg et al. [92]. These authors observed that the quality of nanocrystal dispersion is an important aspect, which affects the quality of the film, making the films more opaque and influencing film strength. 

It is possible to visualize an increase in the mechanical resistance of the films with the incorporation of the nanocrystals dispersion, making them more rigid, but there was an increase in the percentage of deformation for all the films (Table 6). The stiffness in some materials can negatively interfere in the percentage of deformation of the same, the more rigid the material, the more easily it will break. The structure of the material and its composition interfere directly in this parameter. A study [93] showed that chitosan nanocomposites reinforced with chitin whiskers increases the tensile strength and significantly reduces the elongation at break. The work showed tensile strength of 52.23 MPa, and elongation at break of 21.32%.

Although microscopies of the films were not performed in this study, the incorporation of nanocellulose from CS, CH, and CC fibers into the films resulted in strong interactions between the plasticizer and the matrix modifying the mechanical profiles, which can demonstrate the compatibility between the phases. Similar results were identified by Marín–Silva et al. [42] when investigated chitosan nanocomposites with microcrystalline cellulose.

## 5. Conclusions

The results found in the study confirm that the nanocellulose crystals of coconut shell (CS), corn husk (CH), and corncob (CC) fibers incorporated into the chitosan/glycerol films are presented as promising materials for the development of biodegradable composites. The crystallization by acid hydrolysis was favorable, resulting in nanocrystals with great potential to be used as reinforcement due to their size. Although the CS did not present the highest amount of extractable cellulose (12.50%), the highest concentration of nanocellulose in the dispersion after the acid hydrolysis was obtained from this fiber. In this study, wheat bran (WB) was not considered an interesting source of nanocrystals, which may be justified due to the low percentage of cellulose present in this residue.

Significant differences were observed in the properties of the film formulations studied, showing that the source of the nanocrystals is an important parameter to be investigated. The water activity varied from 0.601 (WB Film) to 0.658 (CH Film) and the moisture content from 15.13 (CS Film) to 20.86 (WB Film). The highest values for tensile strength were presented for CC (11.43 MPa) and CS (11.38 MPa) films. Therefore, the films prepared with the nanocrystals dispersion from the CS and CC presented an improvement of the mechanical properties, and the analysis of TEM indicated that both presented good characteristics to be applied in the development of nanobiocomposites. 

## Figures and Tables

**Table 1:** Formulation of films containing nanocrystals of different lignocellulosic sources and control.

Formulations	Chitosan (%, g/100 g)	Acetic Acid (%, g/100 g)	Glycerol (%, g/100 g)	Cellulose Nanocrystals (%, g/100 g)
Control	1.50	1.00	0.15	0.00
CS	1.50	1.00	0.15	5.00
CH	1.50	1.00	0.15	5.00
CC	1.50	1.00	0.15	5.00
WB	1.50	1.00	0.15	5.00

Coconut shell (CS), corn husk (CH), corncob (CC) and wheat bran (WB).

**Table 2:** Mean values of moisture content, water activity, and ash of lignocellulosic fibers (mean ± standard deviation).

Fibers	Moisture (%)	Activity Water	Ash Content (%)
CS	88.7 ± 0.07 ^a^	0.970 ± 0.05 ^a^	5.37 ± 0.10 ^a^
CH	67.1 ± 5.40 ^b^	0.940 ± 0.06 ^b^	0.92 ± 0.12 ^b^
CC	72.7 ± 5.80 ^b^	0.770 ± 0.05 ^c^	3.64 ± 0.26 ^c^
WB	12.4 ± 0.60 ^c^	0.640 ± 0.03 ^d^	4.75 ± 0.27 ^d^

Coconut shell (CS), corn husk (CH), corncob (CC), and wheat bran (WB). Mean ± standard deviation of samples. Values with the same letter in the same column did not present significant differences (*p* < 0.05) by Tukey’s test at 95% confidence (a–d).

**Table 3:** Cellulose, hemicellulose, and lignin contents in natural lignocellulosic fibers (mean ± standard deviation).

Fibers	Cellulose (%)	Hemicellulose (%)	Lignin (%)
CS	47.16 ± 1.24 ^b^	20.71 ± 0.66 ^b^	30.71 ± 0.21 ^a^
CH	24.09 ± 1.13 ^c^	12.99 ± 0.58 ^c^	0.50 ± 0.13 ^c^
CC	52.99 ± 1.79 ^a^	29.72 ± 0.69 ^a^	4.56 ± 1.84 ^b^
WB	10.86 ± 1.25 ^d^	28.88 ± 0.32 ^a^	4.89 ± 0.84 ^b^

Coconut shell (CS), corn husk (CH), Corncob (CC), and wheat bran (WB). Mean ± standard deviation of samples. Values with the same letter in the same column did not present significant differences (*p* < 0.05) by Tukey’s test at 95% confidence (a-d).

**Table 4:** Yield and concentration of cellulose pulp and nanocrystals in the different lignocellulosic sources.

Lignocellulosic Source	CS	CH	CC	WB
Pulp Cellulose (%)	12.50	25.40	38.70	28.00
Nanocellulose (g.10mL^−1^)	0.660	0.050	0.072	NA

Coconut shell (CS), corn husk (CH), corncob (CC), and wheat bran (WB).

**Table 5:** Size of the nanocrystals of different lignocellulosic sources (mean ± standard deviation).

Nanocrystals	L ± sd (nm)	D ± sd (nm)	L/D
CS	254.0 ± 98	6.32 ± 1.02	40.18
CH	298.3 ± 97	7.30 ± 1.20	40.86
CC	302.0 ± 86	8.12 ± 0.96	32.19
WB	-	-	-

Coconut shell (CS), corn husk (CH), corncob (CC), and wheat bran (WB). L = length; D = width and L/D ratio.

**Table 6:** Characterization of nanobiocomposites (mean ± standard deviation).

Film	a_w_±sd	M ± sd (%)	TS ± sd (%)	t ± sd (mm)	σ ± sd (MPa)	ε ± sd (%)
**Control**	0.610±0.01 ^b^	20.75±0.78 ^a^	78.92±0.78 ^d^	0.049±0.02 ^a^	4.08±1.87 ^d^	115.9±4.36 ^e^
**CS Film**	0.600±0.01 ^b^	15.13±0.01 ^c^	84.87±0.01 ^a^	0.040±0.04 ^ab^	11.38±3.53 ^a^	274.2±1.35 ^a^
**CH Film**	0.658±0.02 ^a^	20.24±0.62 ^a^	79.76±0.62 ^c^	0.027±0.01 ^bc^	6.99±4.56 ^c^	155.2±5.13 ^c^
**CC Film**	0.611±0.05 ^b^	18.32±0.90 ^b^	81.68±0.90 ^b^	0.019±0.01 ^c^	11.43±3.58 ^a^	195.2±8.76 ^b^
**WB Film**	0.601±0.01 ^b^	20.86±0.06 ^a^	79.14±0.06 ^cd^	0.027±0.01 ^bc^	4.03 ±1.67 ^b^	141.0±9.09 ^d^

Coconut shell (CS), corn husk (CH), corncob (CC), and wheat bran (WB). Values followed by same letter, in the same column, did not present significant differences (*p* > 0.05) by the Tukey test at 95% confidence (a-d).
